# Pregnancy and acromegaly: clinical outcomes of retrospectively analysed data from the German acromegaly registry

**DOI:** 10.1186/s12958-024-01207-9

**Published:** 2024-04-22

**Authors:** Anke Tönjes, Marleen Würfel, Marcus Quinkler, Ulrich J. Knappe, Jürgen Honegger, Nina Krause-Joppig, Konrad Bacher, Timo Deutschbein, Sylvère Störmann, Jochen Schopohl, Sebastian M. Meyhöfer, the participants of the German Acromegaly Registry, Almuth Meyer, Matthias Gruber, Stefanie Wortmann, Christine Klasen, Wolfram Karges, Frank Demtröder, Hanna Frenzke, Katharina Laubner, Reinhard Finke

**Affiliations:** 1 Medical Department III – Endocrinology, Nephrology, Rheumatology, University of Leipzig Medical Center, Liebigstraße 20, 04103 Leipzig, Germany; 2 Endocrinology in Charlottenburg, Berlin, Germany; 3 Department of Neurosurgery, Johannes Wesling Hospital, Minden, Germany; 4 Department of Neurosurgery, University of Tübingen, Tübingen, Germany; 5 ENDOKRINOLOGIKUM Hannover, Hannover, Germany; 6 Practice for Endocrinology and Diabetes, Stuttgart, Germany; 7 Medicover Oldenburg MVZ, Oldenburg, Germany; 8 Department of Internal Medicine I, Division of Endocrinology and Diabetes, University Hospital, University of Würzburg, Würzburg, Germany; 9 Medizinische Klinik Und Poliklinik IV, LMU Klinikum, Munich, Germany; 10 Medicover Neuroendocrinology, Munich, Germany; 11 Institute of Endocrinology and Diabetes, University of Lübeck, Lübeck, Germany; 12 German Center for Diabetes Research (DZD), Neuherberg, Germany

**Keywords:** Acromegaly, Pregnancy, Dopamine agonists, Somatostatin analogs, Gestational diabetes

## Abstract

**Context:**

Acromegaly is a rare disease caused by excessive growth hormone (GH) secretion, mostly induced by pituitary adenomas. The care of pregnant women with acromegaly is challenging, in part due to existing clinical data being limited and not entirely consistent with regard to potential risks for mother and child.

**Objective:**

To retrospectively examine data on pregnancy and maternal as well as neonatal outcomes in patients with acromegaly.

**Design & methods:**

Retrospective data analysis from 47 pregnancies of 31 women treated in centers of the German Acromegaly Registry.

**Results:**

87.1% of the studied women underwent transsphenoidal surgery before pregnancy. In 51.1% a combination of dopamine agonists and somatostatin analogs were used before pregnancy. Three women did not receive any therapy for acromegaly. During pregnancy only 6.4% received either somatostatin analogs or dopamine agonists. In total, 70.2% of all documented pregnancies emerged spontaneously. Gestational diabetes was diagnosed in 10.6% and gravid hypertension in 6.4%. Overall, no preterm birth was detected. Indeed, 87% of acromegalic women experienced a delivery without complications.

**Conclusion:**

Pregnancies in women with acromegaly are possible and the course of pregnancy is in general safe for mother and child both with and without specific treatment for acromegaly. The prevalence of concomitant metabolic diseases such as gestational diabetes is comparable to the prevalence in healthy pregnant women. Nevertheless, larger studies with more data in pregnant patients with acromegaly are needed to provide safe and effective care for pregnant women with this condition.

## Significance statement

The care of pregnant women with acromegaly is challenging, in part due to existing clinical data being limited and not entirely consistent with regard to potential risks for mother and child. Our registry  belongs to the largest data collections for patients with acromegaly in Europe and provides a significant contribution to the current clinical knowledge. Results of this study support that pregnancies in women with acromegaly are possible and the course of pregnancy is in general safe for mother and child both with and without specific treatment for acromegaly.

## Introduction

Acromegaly is a systemic endocrine disease caused by an overproduction of growth hormone (GH) by pituitary adenoma [1]. It is a rare disease with an incidence of 3–4 in one million per year (270–330 new patients per year in Germany) [2]. The current prevalence in Germany is estimated to be around 5.000 to 10.000 patients. However, it is believed that there are undiagnosed patients with mild forms of acromegaly. Due to the rarity of the disease, the German Acromegaly Registry serves as a valuable database to improve knowledge about the course of disease and therapeutic interventions [2].

As a systemic disease, acromegaly affects multiple organ systems resulting into far-reaching consequences in terms of cardiometabolic parameters such as high blood pressure, lipid profile changes, endothelial dysfunction, diabetes mellitus, heart failure, as well as acral enlargement, sleep apnea, musculoskeletal changes like osteoporosis and arthropathy or the risk of developing cancer [3–5]. The availability of data regarding the course of pregnancy and its outcome for mother and child in women with acromegaly is very limited.

Under physiological conditions and in females with intact GH regulatory circuit, insulin-like growth factor 1 (IGF-1) significantly decreases during the first trimester of pregnancy, whereas GH levels remain stable [6]. However, pituitary-delivered GH cannot cross the placenta. Therefore, there are no expected negative effects on the fetus in patients with acromegaly. In females with acromegaly, a decrease of IGF-1 during the first trimester is described to likely be a result of an increased turnover or decreased production rather than a decrease in pituitary secretion [7]. Moreover, increased circulating levels of estrogens also diminish the hepatic production of IGF-1 during the first trimester. During the second half of pregnancy and by the 15th gestational week, continuous GH secretion from the placenta becomes the main source of circulating GH, while also stimulating liver production of IGF-1 [8, 9]. In pregnant women without acromegaly, placental GH inhibits pituitary GH via a negative feedback mechanism. Placental GH stimulation increases circulating IGF-1 levels with a peak in gestational week 37 and promotes placenta and fetal growth (summarized in [10]). This mechanism is also called “gestational acromegaly” [1]. Due to the aforementioned overlap between physiologic gestational elevation of GH with high circulating placental GH and pregnant females suffering from acromegaly, the separation of both is difficult and leads to the question of which possible therapeutic consequences for pregnancy could thus arise. Several studies observed a stable tumour size during the course of pregnancy under discontinuation of specific medication at the time of confirmation of pregnancy [11]. This led to the assumption that drug treatment can be discontinued in most patients, whereas uncontrolled disease before conception could be a predictor for the development of comorbidities such as diabetes mellitus or hypertension [11].

In the majority of patients acromegaly is associated with hypogonadotropic hypogonadism resulting in a decreased fertility [12, 13]. Nevertheless, previous studies could reveal that therapeutic disease control could result in a recovery of pituitary function and an improvement of fertility [14]. However, spontaneous pregnancy in acromegalic patients with active disease has also been described [12, 15].

In this study, we investigated 47 pregnancies with acromegaly and retrospectively analysed data to address the following questions:

- What medication was taken before and during pregnancy?
- How many patients had surgery or radiation therapy prior to pregnancy?
- How often did a spontaneous pregnancy occur?
- How often do pregnancy complications such as gestational diabetes or hypertension occur?
- How does disease activity change with or without specific acromegaly-specific therapy?
- How many pregnancies have been diagnosed with hypopituitarism?
- How many pregnancies of patients with acromegaly result in healthy newborns?
- What neonatal complications occurred?

## Methods

### Patients

At the time of the study, a total of 61 women included in the German acromegaly register fulfilled the inclusion criteria (pregnancy after diagnosis of acromegaly or pregnancy within five years before diagnosis). The centers caring for these patients were asked to fill a specific questionnaire. For a total of 31 women with 47 documented pregnancies data were included in the analyses. In detail, the period of diagnosis for these women ranged from 1986 to 2016, whereas the period of pregnancy confirmation ranged from 1999 to 2016. The mean (± SD) age at conception was 31.6 ± 5.2 years for the first documented pregnancy. The mean age of the women studied at the initial diagnosis of acromegaly was 27.3 ± 6.4 years. Five of the patients received their diagnosis after their pregnancy, averaging 4.3 years after their first pregnancy.

### Methods

Data were collected via structured questionnaires. Specifically, the questionnaires were sent to centers participating in the German Acromegaly Registry [2]. We obtained data from 36 female patients with documented pregnancy and acromegaly out of 10 centers. Five patients were excluded from the study due to incomplete data on the questionnaire. As a result, 31 patients with complete datasets were eligible for the study. Information collected included age at initial diagnosis, age at conception, mode of conception (spontaneous or by reproductive/ hormonal means), treatment of acromegaly before and during pregnancy as well as associated comorbidities such as the presence of gestational diabetes, gravid hypertension and hypopituitarism. Information was also collected on the course of pregnancy, mode of delivery (natural route or caesarean section), complications during pregnancy and postpartum, miscarriages and anthropometric data of the newborn. Data on GH and IGF-1 levels before, as well as in the first, second and third trimester of pregnancy based on the information provided by the attending physicians about their patients. Descriptive statistical analysis was performed using the statistical software package IBM SPSS statistics version 29.0 for windows.

## Results

**Fig. 1:**
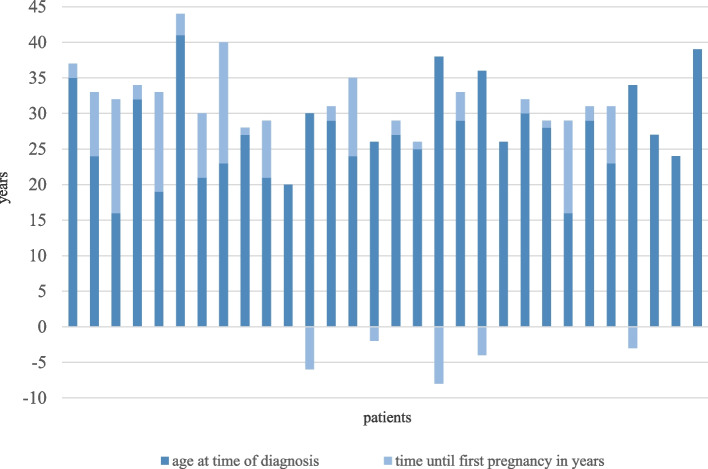
Association between time of diagnosis of acromegaly and first pregnancy in all documented pregnancies (*N* = 31). No anthropometric data were documented for one pregnancy, so only 30 pregnancy cases can be documented here. Y-axis: age in years. X-axis: each column represents data for one pregnant woman. Dark blue represents the age of each patient at the time of diagnosis in years, and the light blue column represents the period of time until the first pregnancy occurred. In five cases, pregnancy occurred before diagnosed acromegaly. Overall, the study included all pregnant women whose diagnosis was up to five years before their first pregnancy

Altogether, of the 47 documented pregnancies, 16 women had one, 14 had two and one women had three pregnancies. The mean age at the time of diagnosis was 27.3 years, whereas the mean age at the first pregnancy was 31.6 years and at each pregnancy was 32.6 years (Fig. 1).

### Treatment before and during pregnancy

Of all pregnant women with acromegaly studied, 27 (87.1% ± 0.34) underwent surgery before their first pregnancy, on average 3.7 months after receiving the diagnosis (Table 1). Only two patients (6.5% ± 0.25) received conventional radiotherapy in addition to surgery before their first pregnancy. One patient received radiotherapy before her third pregnancy. Moreover, of the 31 pregnant women, eight women did not receive any medication for their acromegaly before conception, three of them were in their first pregnancy while five were in their second pregnancy. Moreover, three women who had been pregnant twice only received a specific medication in one of the pregnancies. Together, out of 47 pregnancies, no acromegaly-specific therapy was present in 16 pregnancies at the time of pregnancy confirmation. Overall, of the 47 documented pregnancies, 31.9% received dopamine agonists (DA) and 61.7% received somatostatin analogs (SSA) before getting pregnant, whereas only 6.4% were treated with either SSA or DA during pregnancy. Only 4.3% were treated with Pegvisomant (Peg) before pregnancy, whereas none received Peg during pregnancy. A combination of SSA and DA was seen in 51.1% before conception, whereas 31.9% were treated with SSA and 4.26% with a DA only. Prior to pregnancy, 6.4% did not receive any therapy in the form of surgery, radiation or medications such as DA, SSA, or Peg. Overall, 66% did not receive specific medication with DA, SSA or Peg during pregnancy, 6.4% received either SSA or DA and 21.3% took other medication not specific for acromegaly.

**Table 1:** Maternal outcomes of all documented pregnancies, *N* = 47. Data presented as absolute numbers (N) and percentages (%)

	N	%
**Invasive treatment before pregnancy**		
**Surgical treatment**		
Pregnant women (*N* = 31)	27	87.1
-one pregnancy (*N* = 16)	15	93.6
-two pregnancies (*N* = 14)	11	78.6
-three pregnancies (*N* = 1)	1	100.0
**Conventional radiotherapy**		
Pregnant women (*N* = 31)	3	9.7
-one pregnancy (*N* = 16)	2	12.5
-two pregnancies (*N* = 14)	1	7.1
**Medication before pregnancy**		
Dopamine agonists	15	31.9
Somatostatin analog	29	61.7
GH-receptor antagonist	2	4.3
**Pituitary insufficiency before pregnancy**	3	6.4
**Pregnancy progress**		
Gestational diabetes	5	10.6
Gestational hypertension/ preeclampsia	3	6.4
**Disease activity**		
Increase	3	6.4
Decrease	2	4.3
Unchanged	17	36.2
Missing information	25	53.2
**Tumor size**		
Increase	4	8.5
Decrease	0	0.0
Unchanged	15	31.9
Missing information	28	59.6
**Delivery**		
**Birth procedure**		
Spontaneous delivery	16	34.0
Caesarean section	16	34.0
Missing information	15	31.9
**Birth complications**		
None	41	87.2
Misscarriages	3	6.4
Shoulder dystocia	0	0.0
Others	3	6.4

### Conception and pituitary function

Out of the 47 documented pregnancies, 33 (70.2%) emerged spontaneously. Further, six (12.7%) pregnancies were achieved with the help of reproductive medicine. Out of the pregnancies that resulted with the help of reproductive medicine, three (50.0%) were the result of in-vitro-fertilisation (IVF) only and one of IVF or intracytoplasmic sperm injection (ICSI). Data on pregnancy mode is missing for eight pregnancies (17.0%). Of the three women who received neither therapy for acromegaly in the form of surgery, radiation or drug treatment before pregnancy, all were able to conceive spontaneously and none developed concomitant diseases such as gestational diabetes or pregnancy-associated hypertension.

Moreover, of the three women who received conventional radiotherapy for the tumour, one woman got pregnant spontaneously, two received fertility treatments. Pituitary insufficiency was described for 6.4% of the documented pregnancies before pregnancy confirmation. Whereas all of them received thyroid hormone substitution, only one woman got hydrocortisone therapy. One of them with pituitary insufficiency became pregnant spontaneously, whereas one patient received hormone therapy. In addition, two pregnancies that were accompanied by pituitary insufficiency (66.7%) were associated with gestational hypertension/ preeclampsia. Moreover, one of them with hypopituitarism received therapeutic treatment with DA during pregnancy.

### Maternal outcomes

Gestational diabetes was diagnosed in five of all pregnancies (10.6%). None reported diabetes mellitus or glucose tolerance disorder before pregnancy. Four of them were unipara, one of them had gestational diabetes in her second pregnancy too. Further, in three pregnancies (6.4%), gestational hypertension or preeclampsia occurred. None of the pregnant women developed postpartum cardiomyopathy. In total, the patients reported no complications during 39 pregnancies (83.0%). Out of the patients on DA, SSA and Peg therapy during pregnancy, in three of those six (50.0%) gestational diabetes was diagnosed. In none of all investigated pregnancies a tumour reduction could be observed, whereas in 8.5% a progression in size was recognized. However, for 59.6% no information was available. Out of all investigated pregnancies, 16 (34.0%) delivered spontaneously, 16 (34.0%) by caesarean section and for 15 (31.9%) patients no information was available. In total, 87.9% had a delivery without any complications.

### Neonatal outcomes

No preterm birth was reported (Table 2). One pregnant woman reported that she had voluntarily terminated her pregnancy in the 8th week of her first pregnancy. Two other women reported an abortion during their second pregnancy. In one newborn a malformation in the sense of an additional 6th finger was registered. In total, 42.6% of newborns had normal birth weight, while 4.3% each were overweight (> 4.200 g) or underweight (< 2.800 g). Median birth weight was 3498 g, whereas mean birth length was 51.6 cm.

**Table 2:** Neonatal outcomes of all documented pregnancies, *N* = 47. Data presented as absolute numbers (N) and percentages (%)

	**N**	**%**
Time of delivery		
Preterm	0	0.0
Born at term	47	100.0
Anthropometric data		
Normal birth weight (2800-4200 g)	20	42.6
Birth weight > 4200 g	2	4.3
Birth weight < 2800 g	2	4.3
Missing information	23	48.9

## Discussion

The aim of this retrospective study was to analyse maternal and neonatal outcomes of 47 pregnancies in women with acromegaly.

In our cohort, the majority of pregnancies arose spontaneously. Although acromegaly is widely discussed to be associated with infertility, spontaneous pregnancy is possible [12]. In discussing our results, it is important to note that of the pregnancies studied, pituitary insufficiency was diagnosed only in three patients before pregnancy. However, we have no information about the number of women with an unfulfilled wish to have children. The course of pregnancy in women with known hypopituitarism is associated with a higher risk for complications [16–18]. In our study, case numbers are very small and data are rather descriptive, but taken together they suggest that the presence of pituitary insufficiency and the quality of disease control play a role in the mode of conception and thus provide considerable additional value to the existing literature [19].

Regarding therapeutic options for the treatment of acromegaly, three classes of drugs (DA, SSA and GH receptor ligands such as Peg) which cross the placenta play an important role. Prior to conception, in our study 23 women were treated with at least one medication. During pregnancy, 66.0% of women with acromegaly did not receive specific medication. In our cohort, those pregnancies in patients treated with either DA or SSA during pregnancy were uneventful. These results are consistent with other studies [10, 20, 21]. Most patients suffering from acromegaly did not develop an increase in tumour size during pregnancy [22]. Furthermore, several studies report stable results of visual field investigations even without treatment during pregnancy [23, 24]. Data are not entirely consistent and there are also reports of an increase in tumour sizes, especially in those patients, who obtained the diagnosis during pregnancy [25, 26]. The guidelines of the “Endocrine Society” recommend a discontinuation of drug medication like SSA and Peg approximately two months before conception and if necessary the change to the SSA octreotide until conception [27]. Furthermore, they clarify that the medication for acromegaly should be used very cautiously during pregnancy [27]. In our cohort, 87.2% did not receive specific drugs like SSA, DA or Peg during pregnancy. The majority of the women did not show any progression of tumour size or any other adverse events. Although those who received acromegaly-specific drugs such as somatostatin analogs delivered by caesarean section, they did not report any complications during pregnancy or fetal development. Despite the limited data available on the safety and teratogenicity of acromegalic-specific drugs during pregnancy, previous reports have shown that treatment with somatostatin analogs in particular is safe and well tolerated during pregnancy [28, 29]. Case studies show that treatment with the somatostatin analog octreotide is generally safe for mother and child [28, 29]. Overall, acromegaly-specific medication during pregnancy does not appear to have teratogenic effects, but is generally limited to specific cases associated with e.g. visual impairment or tumor expansion (reviewed in [10]).

Acromegaly can be associated with many concomitant diseases such as impaired glucose tolerance or diabetes mellitus. Petrossians et al. presented that at the time of diagnosis, diabetes mellitus was prevalent in 27.5% of the patients with acromegaly [30]. In pregnant women with acromegaly, the prevalence of gestational diabetes is scarcely investigated. However, Vialon et al. studied 14 pregnancies in 11 women, who had no diabetes mellitus before conception [31]. During the course of these pregnancies, they diagnosed gestational diabetes in 50% of the investigated pregnancies that was confirmed on either fasting blood glucose or oral glucose tolerance test. Further, Caron et. al detected diabetes mellitus in 6.8% of 59 pregnancies and stated that there is a higher prevalence of gestational diabetes in acromegalic women compared to the general French population of pregnant women [25]. In comparison, data from pregnant women without acromegaly in Germany show that the prevalence of gestational diabetes generally increased from 4.6% to 7.3% between 2013 and 2019 [32]. In our present study we found that gestational diabetes was more common in our cohort than in the pregnant population without acromegaly, as 10.6% of all pregnancies documented here were associated with gestational diabetes. All women suffering from gestational diabetes delivered by caesarean section, but neither postpartum diseases nor anomalies of the newborn were detected. This is in line with results of other retrospective studies presenting a normal gestational course of pregnant women with acromegaly [24, 25]. Moreover, we could observe gestational hypertension in 6.4%. All women could deliver spontaneously and no peri- or postpartum complications were detected, which is in line with former clinical outcomes presenting no increase of fetal or maternal complications regarding gestational hypertension [24, 33]. In total, hypertension and gestational diabetes play a relevant role as major clinic elements and cardiovascular risk factors that should be given particular attention during pregnancy in acromegalic women. As far as we currently know, data from pregnant women with acromegaly do not show that there is a higher rate of cardiovascular complications affecting fetal and maternal outcomes compared with the general pregnant population without acromegaly (reviewed in [10]). Nevertheless, it is of great importance to maintain sensibility to detect gestational diabetes and hypertension. Additionally, it is necessary to continue collecting data from pregnant women with acromegaly to elicit valid conclusions in the future about the associations of acromegaly, metabolic comorbidities as well as their impact and consequences on pregnancy.

The major shortcomings of our study are its retrospective nature and the lack of many data. Especially we had only information about disease activity in 53.2% of the 47 pregnancies, no visual field data and no information on tumor size during the course of pregnancy. However, our study provides the largest cohort regarding pregnancy in acromegalic women so far.

## Conclusion

Overall, the care of pregnant women with acromegaly represents a special clinical constellation and involves complexities when considering acromegaly treatment alongside potential coexisting conditions. Overall, the data of pregnant women with acromegaly are very limited and future data collection and observation of more of this patient population is needed. However, the data obtained so far indicate that spontaneous pregnancy is possible if disease control is achieved prior to conception. In addition, drug therapy should be discontinued at the time pregnancy is established and used only in exceptional individual situations for tumour or symptom control. Depending on the available study data, an increased, decreased or equal prevalence of metabolic concomitants such as diabetes mellitus during pregnancy with acromegaly could be detected. To assess potential factors influencing the prevalence of metabolic concomitant diseases, several anthropometric data, such as age, weight and BMI, as well as diagnostic tools for early detection of glucose tolerance disorders are needed. Nevertheless, the data collected here, together with previous retrospective studies, show that pregnancy in patients with acromegaly is often without complications and with safe peri- and postpartum outcome for mothers.

## Data Availability

The data is available on request.
